# Lower dietary folate intake increases the risk of autoimmune thyroiditis

**DOI:** 10.3389/fnut.2025.1595825

**Published:** 2025-06-19

**Authors:** Lijun Chen, Changjian Yan, Yingxuan Lin, Liqun Huang, Chunling Huang, Zhida Wei, Hongli Lin, Xiaohong Wu, Ruhai Lin, Zhengrong Jiang, Huibin Huang

**Affiliations:** ^1^Department of Endocrinology, The Second Affiliated Hospital of Fujian Medical University, Quanzhou, Fujian Province, China; ^2^Department of Hematology, The Second Affiliated Hospital of Fujian Medical University, Quanzhou, Fujian Province, China; ^3^School of Clinical Medicine, Fujian Medical University, Fuzhou, Fujian Province, China; ^4^Department of Nuclear Medicine, The Second Affiliated Hospital of Fujian Medical University, Quanzhou, Fujian Province, China; ^5^Department of Electrocardiogram, The Second Affiliated Hospital of Fujian Medical University, Quanzhou, Fujian Province, China; ^6^Information Center, The Second Affiliated Hospital of Fujian Medical University, Quanzhou, Fujian Province, China

**Keywords:** autoimmune thyroiditis, dietary folate intake, risk prediction model, cross-sectional study, nutritional epidemiology

## Abstract

**Background:**

Autoimmune thyroid diseases (AITDs) are a group of organ-specific autoimmune disorders resulting from the loss of immune tolerance, with autoimmune thyroiditis (AIT) being the most common phenotype. In recent years, folate, an essential nutrient, has been associated with the onset of various autoimmune diseases. However, the relationship between dietary folate intake and AIT remains unclear.

**Objective:**

This study seeks to explore the possible link between folate consumption and AIT.

**Methods:**

This study is based on the 2009–2010 National Health and Nutrition Examination Survey (NHANES) data to analyze the connection between folate consumption and the risk of AIT. A total of 2037 participants were included in the study. Based on TPOAb or TgAb levels, participants were classified into the AIT group (*n* = 144) and the non-AIT group (*n* = 1893), and clinical variables were compared between these two groups. Univariate and multivariate logistic regression models were employed to examine the relationship between AIT risk and various factors, including demographics, complete blood count, blood biochemistry parameters, thyroid function test, urinary iodine concentration, as well as intakes of vitamin B12 and folate. A diagnostic model for AIT was constructed using dietary folate intake, TSH, age, sex, urinary iodine concentration, and vitamin B12 intake.

**Results:**

The analysis results indicate that the dietary folate intake of AIT patients was significantly lower than that of the non-AIT group (356.7 ± 172.4 vs. 396.1 ± 200.3mcg/day, *p* < 0.05). Participants in the high dietary folate intake group showed a 52% lower risk of AIT compared to the low-intake group (OR = 0.48, 95%CI: 0.33–0.71, *p* < 0.001) in the univariate analysis. This association remained significant after multivariable adjustment (OR = 0.53, 95%CI: 0.35–0.80, *p* = 0.003).

**Conclusion:**

This cross-sectional study is the first to explore the association between dietary folate consumption and the likelihood of developing AIT. The results suggest that lower dietary folate intake may be an independent factor contributing to AIT. It may provide new insights for the development of future dietary prevention strategies for AIT.

## Introduction

The autoimmune thyroid diseases (AITDs) are the most common organ-specific autoimmune disorders, including hyperthyroidism (Graves’ disease) and autoimmune thyroiditis (AIT) (1). The primary characteristic of AITDs is lymphocytic infiltration within the thyroid gland (2). Among them, AIT is represented by Hashimoto’s thyroiditis (HT), with an incidence rate of approximately 0.3–1.5 per 1,000 people per year. The disease occurs 4–10 times more frequently in women than in men (3). AIT is a leading factor in primary hypothyroidism, primarily diagnosed by relying on positive titers of serum anti-thyroid peroxidase antibodies (TPOAb) and anti-thyroglobulin antibodies (TgAb), along with diffuse hypoechoic findings on thyroid ultrasound. Studies have shown that approximately 80–90% of AIT patients have positive serum TPOAb, with a sensitivity of up to 90%. Additionally, 60–80% of patients show positive serum TgAb, with a diagnostic sensitivity ranging from 30 to 50% (3).

Research has shown that the presence of serum TPOAb and/or TgAb is strongly linked to negative pregnancy outcomes, including miscarriage, preterm birth, gestational diabetes, and other obstetric complications (4). In approximately 20% of patients, AITDs are associated with other organ-specific or systemic autoimmune diseases (3).

AITDs are mediated by B cells and T cells, primarily resulting from the loss of immune tolerance and immune attack on thyroid tissue (2, 5). The pathogenesis of AIT is generally believed to result from the interaction of genetic susceptibility, environmental factors and epigenetic effects. Genetic factors include polymorphisms in immune-regulatory genes (such as HLA-DR3, PTPN22, CD40, and CTLA4) and thyroid-specific genes (such as TSHR, Tg, and TPO genes) (1). Environmental factors, such as viral infections, alterations in the gut microbiota, medications (e.g., interferon-*α*), and deficiencies in nutrients, are all associated with the development of AIT (6–12). Epigenetic factors, such as DNA methylation and non-coding RNAs, may promote the onset of AIT by regulating immune cell function and thyroid autoimmune responses (13–19). Additionally, oxidative stress is also considered an important factor in the pathogenesis of AIT (20).

Nutritional factors have been shown to closely influence the risk of AIT. Studies have shown that adequate intake or supplementation of vitamin D, selenium, vitamin A, vitamin B12, iron, magnesium, and vitamin E can help reduce the risk of AIT or regulate the levels of TPOAb and TgAb (6, 21–25). However, existing clinical guidelines do not provide specific dietary recommendations for autoimmune thyroid disorders.

Folate (vitamin B9) is a water-soluble vitamin that plays a role in one-carbon metabolism and amino acid metabolism. Folate helps lower homocysteine (Hcy) levels, with studies showing that folate supplementation can reduce Hcy levels by 25% (26). Folate deficiency can lead to elevated Hcy levels, increasing the risk of cardiovascular diseases and certain autoimmune disorders (27). Hypothyroidism is often associated with elevated Hcy levels (28). Additionally, studies have found that patients with hypothyroidism have lower folate levels, and their hyperhomocysteinemia (HHcy) is associated with altered folate status (29). Folate deficiency is associated with the risk of various diseases, such as anemia, coronary heart disease, and cancer (26–29). Studies have also found that 16.3% of patients with positive anti-thyroid antibodies have anemia, with 1.1% having folate deficiency (30). However, the relationship between folate and thyroid metabolism remains unclear and requires further investigation.

This study, based on data from the 2009–2010 National Health and Nutrition Examination Survey (NHANES), aims to explore the potential association between dietary folate intake and AIT.

## Materials and methods

### Data analysis from NHANES

#### Study population

NHANES conducted by the National Center for Health Statistics (NCHS) of the Centers for Disease Control and Prevention (CDC),^1^ is a nationwide cross-sectional study that assesses the health, laboratory tests, and dietary nutritional status of both adults and children in the United States. Participants provide information about their demographic and socioeconomic characteristics, as well as health status, through standardized questionnaires. The survey results are commonly used to assess the dietary nutritional status, prevalence of diseases, and associated risk factors in a representative sample of the US population. These findings guide the work and decision-making of government agencies, community organizations, non-profit groups, and businesses in public health and healthcare. Each NHANES study protocol is reviewed and approved by the Institutional Review Board (IRB) of the NCHS, with informed consent obtained from all participants.

**Figure 1:**
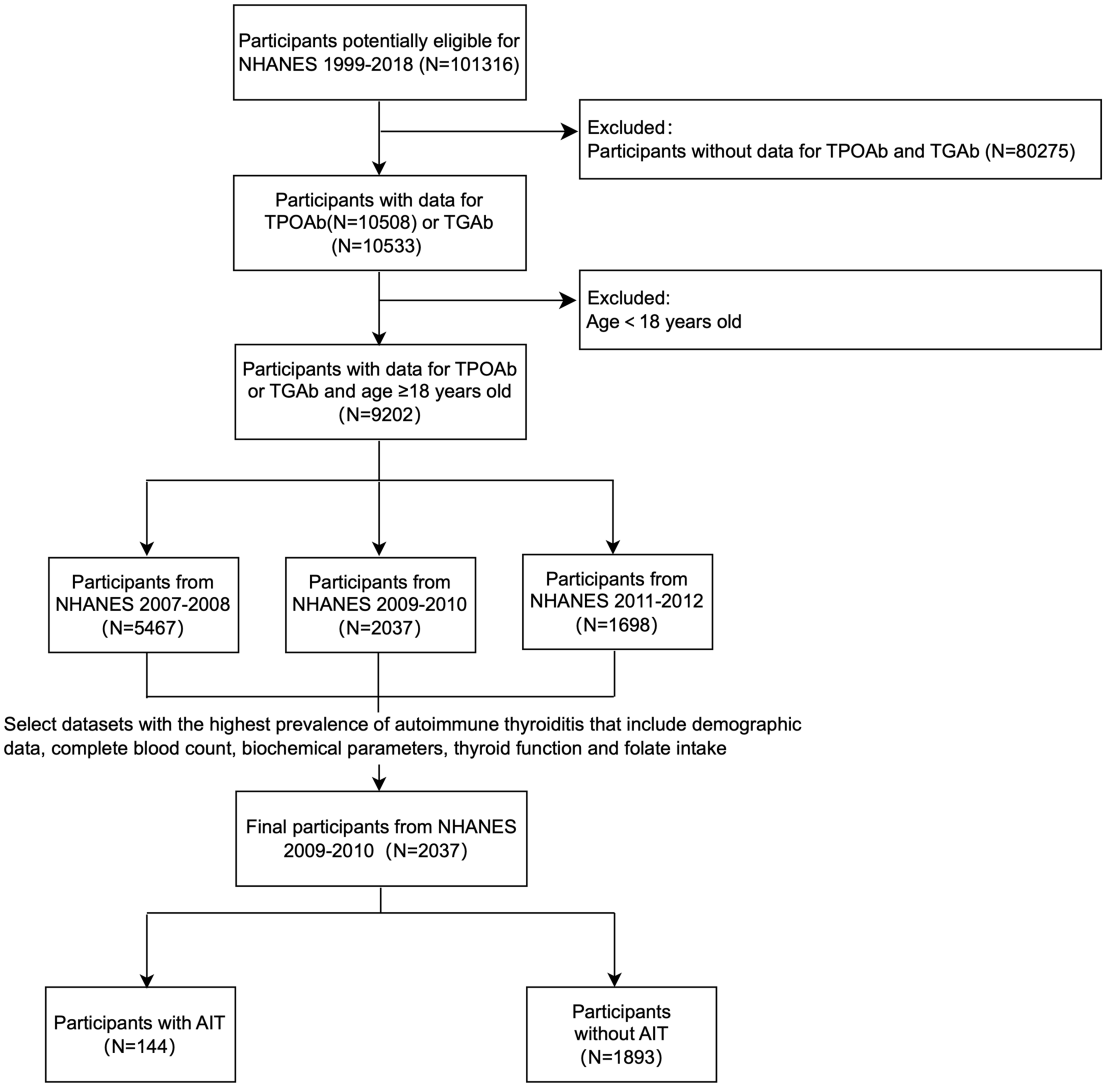
Participant flow diagram.

The research utilized a cross-sectional approach, combining information through individual participants’ distinct “SEQN” identifiers. Using NHANES records from 1999 to 2018, we included 101,316 participants who had complete demographic characteristics, laboratory test results (complete blood count, blood biochemical parameters, thyroid function tests), and dietary folate intake data. The exclusion criteria were as follows: ① Participants with missing data for both TPOAb and TgAb tests. ② Participants under the age of 18. Only the NHANES 2007–2008, NHANES 2009–2010, and NHANES 2011–2012 datasets met the inclusion criteria. AIT was defined as TPOAb ≥ 9 IU/mL or TgAb ≥ 4 IU/mL. The NHANES 2009–2010 dataset, which had the highest prevalence of AIT (7.1%), was selected for the study, including a total of 2037 participants. These were divided into a non-AIT group (1893 participants) and an AIT group (144 participants; Figure 1).

### Thyroid profile

After venous blood samples were obtained, thyroid function and specific autoantibody levels were assessed following standard procedures. Free thyroxine (fT4) was measured via a two-step enzyme-linked immunosorbent assay (ELISA), with a reference range of 0.6–1.6 ng/dL. Thyroid-stimulating hormone (TSH) was analyzed using a dual-point immunoenzymatic ‘sandwich’ method, with a reference range of 0.34–5.6 mIU/mL. TgAb (0–4 IU/mL) and TPOAb (0–9 IU/mL) were assessed using the Beckman Access2 immunoassay system (31).

### Diagnostic criteria

TPOAb positivity was defined as a level ≥ 9 IU/mL, while TgAb positivity was defined as a level ≥ 4 IU/mL. The diagnosis of AIT was determined by the presence of elevated TPOAb or TgAb levels.

### Dietary folate assessment

In NHANES, folate consumption from food and supplements was assessed using two separate 24-h dietary recall sessions. The initial interview was performed in person by trained interviewers at community health education centers, while the follow-up interview occurred by phone or mail 3 to 10 days later. To minimize bias, the study used the average total folate intake from both recall interviews. Additional details regarding the dietary survey methodology can be found in the “NHANES Dietary Interviewer Procedures Manual” (32). The analysis involved transforming average folate consumption into a dichotomous variable. The cutoff point for this conversion was established through ROC curve evaluation. Using the pROC library’s coordinate function, we identified 271.75 mcg as the most effective dividing value. Participants were subsequently categorized into high and low intake groups based on this threshold. This critical value was chosen by carefully considering both detection rate and precision metrics. Our approach sought to achieve equilibrium between correctly identifying true cases and reducing incorrect exclusions, ultimately enhancing the model’s predictive capability.

### Variables

We have collected 45 variables, as detailed in Supplementary Table S1.

### Statistical analysis

Normally distributed data are presented as mean ± standard deviation, with group comparisons conducted using an independent t-test. Non-normal data are expressed as median (range) and analyzed via the rank-sum test. Categorical variables are reported as percentages (%). For ordinal categorical variables, the rank-sum test was applied, while the chi-square test (χ^2^ test) was used for nominal variables. A *p* value less than 0.05 was considered statistically significant.

Multivariate logistic stepwise regression was used to identify risk factors and construct the corresponding risk model. All statistical analyses were performed using R software version 4.4.1.^2^ The plots were generated using the ggplot2 package.^3^ A *p* value less than 0.05 was considered statistically significant.

## Results

### Baseline characteristics

This study is based on the NHANES database, selecting 2037 participants from the 2009–2010 survey cycle as the study subjects. Participants were aged between 18 and 80 years, with the oldest participant being 80 years old. All participants underwent testing for TPOAb and TgAb. A total of 144 cases of AIT were diagnosed, with an overall prevalence of 7.1%. The clinical characteristics of patients in the AIT group and non-AIT group were further analyzed for differences (Table 1).

**Table 1:** Baseline characteristics of AIT patients and non-AIT patients.

Category	Characteristics	Levels	Overall	non-AIT group	AIT group	*p*-value
	n		2037	1893	144	
Demographics	Age, year [mean (SD)]		**48.239 (18.525)**	**47.911 (18.509)**	**52.549 (18.256)**	**0.0038**
	Gender (%)	Female	**1,038 (50.96)**	**945 (49.92)**	**93 (64.58)**	
		Male	**999 (49.04)**	**948 (50.08)**	**51 (35.42)**	**0.0009**
	Height, cm [mean (SD)]		**167.383 (10.283)**	**167.577 (10.233)**	**164.837 (10.639)**	**0.0021**
	Weight, kg [mean (SD)]		81.431 (21.547)	81.562 (21.487)	79.699 (22.327)	0.3205
	BMI, kg/m^2^ [mean (SD)]		28.989 (6.927)	28.967 (6.904)	29.280 (7.244)	0.6036
	Race (%)	Mexican American	392 (19.24)	366 (19.33)	26 (18.06)	0.1029
		Other Hispanic	209 (10.26)	186 (9.83)	23 (15.97)	
		Non-Hispanic White	956 (46.93)	886 (46.80)	70 (48.61)	
		Non-Hispanic Black	355 (17.43)	336 (17.75)	19 (13.19)	
		Other Race	125 (6.14)	119 (6.29)	6 (4.17)	
Complete Blood Count	WBC, 1000 cells/uL [mean (SD)]		7.188 (2.524)	7.182 (2.551)	7.266 (2.124)	0.7013
	HGB, g/dL [mean (SD)]		**14.079 (1.489)**	**14.102 (1.485)**	**13.777 (1.507)**	**0.012**
	RBC, 1000 cells/uL [mean (SD)]		**4.594 (0.515)**	**4.602 (0.514)**	**4.496 (0.519)**	**0.0181**
	NE, 1000 cells/uL [mean (SD)]		4.255 (2.110)	4.250 (2.133)	4.321 (1.777)	0.6971
	LYM, 1000 cells/uL [mean (SD)]		2.150 (0.890)	2.151 (0.902)	2.145 (0.703)	0.94
	MO, 1000 cells/uL [mean (SD)]		0.538 (0.187)	0.538 (0.185)	0.540 (0.216)	0.8926
	EO, 1000 cells/uL [mean (SD)]		0.204 (0.199)	0.203 (0.202)	0.220 (0.163)	0.3237
	PLT, 1000 cells/uL [mean (SD)]		237.916 (61.511)	237.492 (61.545)	243.542 (60.989)	0.2585
	RDW (%)		**12.924 (1.347)**	**12.907 (1.346)**	**13.142 (1.339)**	**0.0447**
Blood Biochemistry Parameters	ALB, g/dL [mean (SD)]		4.241 (0.343)	4.242 (0.344)	4.229 (0.320)	0.6603
	GLO, g/dL [mean (SD)]		2.952 (0.490)	2.947 (0.488)	3.018 (0.510)	0.0937
	TP, g/dL [mean (SD)]		7.193 (0.495)	7.189 (0.497)	7.247 (0.464)	0.1735
	CHOL, mmol/L [mean (SD)]		192.925 (42.127)	192.843 (42.239)	194.000 (40.760)	0.7508
	Bicarbonate, mmol/L [mean (SD)]		25.477 (2.149)	25.460 (2.138)	25.701 (2.281)	0.1939
	SCR, mg/dL [mean (SD)]		0.907 (0.455)	0.905 (0.400)	0.937 (0.910)	0.4038
	SGL, mg/dL [mean (SD)]		101.345 (37.203)	100.900 (36.201)	107.188 (48.248)	0.0506
	STB, mg/dL [mean (SD)]		0.758 (0.292)	0.760 (0.294)	0.731 (0.267)	0.251
	TG, ng/mL [mean (SD)]		151.339 (124.018)	151.673 (125.574)	146.958 (101.683)	0.6602
	UA, mg/dL [mean (SD)]		5.455 (1.456)	5.470 (1.468)	5.253 (1.278)	0.0838
	NA, mmol/L [mean (SD)]		139.361 (2.139)	139.374 (2.104)	139.188 (2.564)	0.3141
	KS, mmol/L [mean (SD)]		3.982 (0.329)	3.982 (0.332)	3.991 (0.286)	0.7452
	CL, mmol/L [mean (SD)]		103.984 (2.886)	104.010 (2.842)	103.646 (3.404)	0.1444
	BUN, mg/dL [mean (SD)]		13.203 (5.889)	13.170 (5.951)	13.625 (4.991)	0.372
	TCA, mg/dL [mean (SD)]		9.441 (0.361)	9.440 (0.361)	9.444 (0.356)	0.898
	AST, U/L [mean (SD)]		26.702 (15.335)	26.605 (15.124)	27.972 (17.888)	0.3025
	ALT, U/L [mean (SD)]		26.247 (19.942)	26.210 (19.838)	26.736 (21.322)	0.7604
	LDH, U/L [mean (SD)]		132.522 (31.839)	132.338 (32.177)	134.931 (26.989)	0.3464
	GGT, U/L [mean (SD)]		28.794 (34.904)	28.970 (35.600)	26.479 (23.942)	0.4092
Thyroid Function Test	TSH, mIU/L [mean (SD)]		**1.959 (3.679)**	**1.851 (2.843)**	**3.390 (9.163)**	**<0.0001**
	fT3, pg./mL [mean (SD)]		3.190 (0.707)	3.195 (0.720)	3.116 (0.486)	0.196
	fT4, pmol/L [mean (SD)]		10.387 (2.210)	10.361 (2.088)	10.734 (3.428)	0.052
	TT3, nmol/L [mean (SD)]		1.769 (0.391)	1.771 (0.386)	1.748 (0.450)	0.4864
	TT4, ug/dL [mean (SD)]		8.104 (1.700)	8.091 (1.646)	8.267 (2.298)	0.2356
	TPOAb, IU/mL [mean (SD)]		**20.633 (87.540)**	**15.846 (75.902)**	**94.304 (177.193)**	**<0.0001**
	TgAb, IU/mL [mean (SD)]		**10.182 (92.194)**	**3.913 (49.184)**	**115.192 (315.445)**	**<0.0001**
	TG,ng/mL [mean (SD)]		15.713 (28.112)	15.552 (27.174)	17.821 (38.423)	0.3506
Urinary iodine concentration	UIC, ug/L [mean (SD)]		231.124 (645.653)	233.981 (666.808)	192.707 (206.036)	0.4673
Vitamin B12 Intake	Vitamin B12 intake, mcg [mean (SD)]		0.949 (1.874)	0.955 (1.886)	0.867 (1.727)	0.6156
Folate Intake	Total dietary folate intake, mcg [mean(SD)]	Low group(<271.75mcg)	**488 (28.72)**	**435 (27.60)**	**53 (43.09)**	**0.0004**
		High group(≥271.75mcg)	**1,211 (71.28)**	**1,141 (72.40)**	**70 (56.91)**	
	Total folate supplement intake (DFE), mcg DFE [mean (SD)]		865.020 (573.267)	872.804 (589.894)	751.091 (184.715)	0.336

n, number of patients; BMI, Body mass index; WBC, White blood cell count; HGB, Hemoglobin; RBC, Red blood cell count; NE, Segmented neutrophils num; LYM, Lymphocyte number; MO, Monocyte numbe; EO, Eosinophils number; PLT, Platelet count; RDW, Red cell distribution width; ALB, Albumin; GLO, Globulin; TP, Total protein; CHOL, Cholesterol; SCR, Creatinine; SGL, Glucose, serum; STB, Total bilirubin; TG, Triglycerides; UA, Uric acid; NA, Sodium; KS, Potassium; CL, Chloride; BUN, Blood urea nitrogen; TCA, Total calcium; AST, Aspartate aminotransferase; ALT, Alanine aminotransferase; LDH, Lactate dehydrogenase; GGT, Gamma glutamyl transferase; TSH, Thyroid stimulating hormone; fT3, Triiodothyronine (T3), free; fT4, Thyroxine, free; TT3, Triiodothyronine, total; TT4, Thyroxine, total; TPOAb, Thyroid perioxidase antibodies; TgAb, Thyroglobulin antibodies; TG, Thyroglobulin; UIC, Urinary iodine concentration; Continuous variables are expressed as mean ± standard deviation, while categorical variables are presented as percentages. Data were analyzed using χ2 tests for the categorical and ANOVA analysis for continuous variables. The bold values indicate statistical significance (*p*-value < 0.05).

*Demographics*: The average age of the non-AIT group was 47.911 ± 18.509 years, whereas the AIT group was significantly older, with an average age of 52.549 ± 18.256 years. The proportion of female patients in the AIT group (64.58%) was higher than that in the non-AIT group (49.92%). The mean height of the AIT group (164.837 ± 10.639 cm) was significantly lower than that of the non-AIT group (167.577 ± 10.233 cm). The *p* values for all comparisons were less than 0.05.

*Complete blood count*: Hemoglobin (HGB) levels showed statistically significant differences among groups (*p* = 0.012). The overall cohort averaged 14.079 ± 1.489 g/dL, with the non-AIT group maintaining higher levels (14.102 ± 1.485 g/dL) compared to the AIT group (13.777 ± 1.507 g/dL). Red blood cell count (RBC) was significantly lower on average in the AIT group (4.496 ± 0.519 × 10^3^ cells/μL) compared to the non-AIT group (4.602 ± 0.514 × 10^3^ cells/μL; *p* = 0.0181). The red blood cell distribution width (RDW) was higher in the AIT group (13.142% ± 1.339%) compared to the non-AIT group (12.907% ± 1.346%; *p* = 0.0447). The above differences were statistically significant.

*Blood biochemical indicators*: No statistically significant differences were observed for any of the variables.

*Thyroid function tests*: The AIT group exhibited a significantly higher mean TSH level (3.390 ± 9.163 mIU/L) compared to the non-AIT group (1.851 ± 2.843 mIU/L), with all comparisons showing *p* values below 0.05.

*Urinary iodine concentration*: Urinary iodine concentration (UIC) analysis revealed no statistically significant intergroup differences (*p* = 0.467). The overall mean was 231.1 ± 645.7 μg/L, with the non-AIT group showing 234.0 ± 666.8 μg/L versus 192.7 ± 206.0 μg/L in the AIT group.

*Vitamin B12 Intake*: Vitamin B12 intake analysis demonstrated comparable levels across groups (*p* = 0.616). The cohort-wide average was 0.949 ± 1.874 mcg, with minimal variation between non-AIT (0.955 ± 1.886 mcg) and AIT groups (0.867 ± 1.727 mcg).

**Figure 2:**
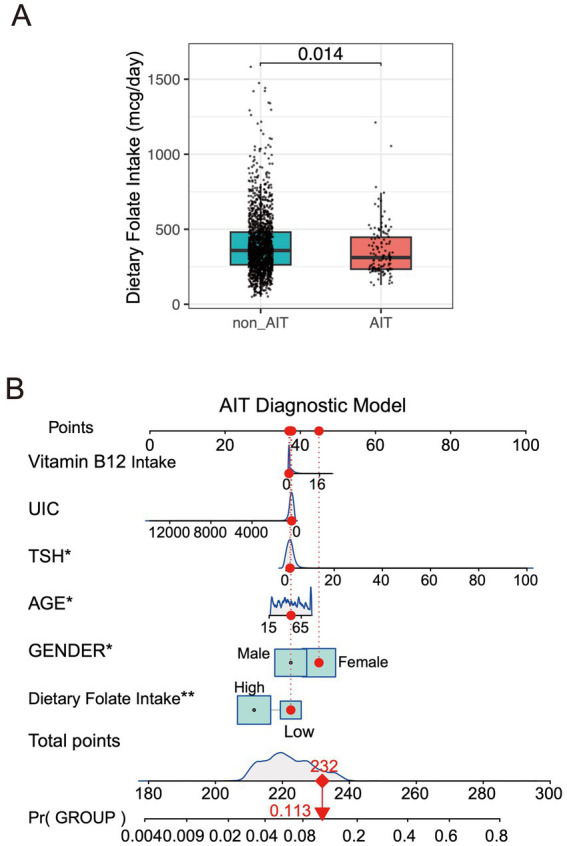
**(A)** Comparison of dietary folate intake (mcg/day) between the non-AIT group and the AIT group. **(B)** AIT diagnostic model.

*Folate Intake*: The mean dietary folate intake in the AIT group (356.7 ± 172.4 mcg) was lower than that in the non-AIT group (396.1 ± 200.3 mcg; *p* < 0.05; Figure 2A). In the low-intake group (< 271.75 mcg), the proportion of AIT patients was significantly higher than in the non-AIT group (43.09% vs. 27.60%), whereas the high-intake group (≥ 271.75 mcg) showed the opposite trend (56.91% vs. 72.40%), suggesting that dietary folate may have a protective effect. However, no significant difference was observed in supplemental folate intake (DFE) between the two groups (*p* = 0.336), although the mean value was slightly lower in the AIT group (751.1 ± 184.7 vs. 872.8 ± 589.9 mcg DFE).

### Independent risk factors for AIT

This study investigated factors linked to AIT risk using both univariate and multivariate logistic regression analyses (Table 2).

**Table 2:** Univariate logistic regression analysis and multivariate logistic regression analysis in AIT patients and non-AIT patients.

Category	Characteristics	Levels	OR (univariable)	OR (multivariable)
Demographics	Age, year		**1.01 (1.00–1.02, *p* = 0.028)**	**1.01 (1.00–1.02, *p* = 0.040)**
	Gender (%)	Female		
		Male	**1.87 (1.25–2.80, *p* = 0.002)**	**1.63 (1.07–2.47, *p* = 0.021)**
	Height, cm		0.98 (0.96–1.00, *p* = 0.054)	
	Weight, kg		1.00 (0.99–1.01, *p* = 0.513)	
	BMI, kg/m^2^		1.00 (0.98–1.03, *p* = 0.750)	
	Race (%)	Mexican American		
		Other Hispanic	1.78 (0.90–3.51, *p* = 0.095)	
		Non-Hispanic White	1.05 (0.62–1.79, *p* = 0.849)	
		Non-Hispanic Black	0.80 (0.40–1.60, *p* = 0.532)	
		Other Race	0.86 (0.31–2.36, *p* = 0.770)	
Complete Blood Count	WBC, 1000 cells/uL		0.99 (0.91–1.07, *p* = 0.793)	
	HGB, g/dL		0.91 (0.80–1.03, *p* = 0.134)	
	RBC, 1000 cells/uL		0.74 (0.50–1.08, *p* = 0.114)	
	NE, 1000 cells/uL		1.00 (0.91–1.09, *p* = 0.940)	
	LYM, 1000 cells/uL		0.96 (0.76–1.21, *p* = 0.721)	
	MO, 1000 cells/uL		0.77 (0.27–2.22, *p* = 0.635)	
	EO, 1000 cells/uL		0.84 (0.29–2.43, *p* = 0.746)	
	PLT, 1000 cells/uL		1.00 (1.00–1.00, *p* = 0.701)	
	RDW(%)		1.12 (1.00–1.24, *p* = 0.051)	
Blood Biochemistry Parameters	ALB, g/dL		1.39 (0.78–2.47, *p* = 0.267)	
	GLO, g/dL		1.16 (0.80–1.68, *p* = 0.447)	
	TP, g/dL		1.34 (0.92–1.94, *p* = 0.124)	
	CHOL, mmol/L		1.00 (1.00–1.01, *p* = 0.121)	
	Bicarbonate, mmol/L		1.04 (0.95–1.14, *p* = 0.341)	
	SCR, mg/dL		1.12 (0.74–1.69, *p* = 0.606)	
	SGL, mg/dL		1.00 (1.00–1.01, *p* = 0.267)	
	STB, mg/dL		0.91 (0.47–1.76, *p* = 0.789)	
	TG, ng/mL		1.00 (1.00–1.00, *p* = 0.676)	
	UA, mg/dL		0.92 (0.80–1.06, *p* = 0.237)	
	NA, mmol/L		1.00 (0.91–1.09, *p* = 0.967)	
	KS, mmol/L		1.13 (0.63–2.02, *p* = 0.686)	
	CL, mmol/L		0.98 (0.91–1.05, *p* = 0.516)	
	BUN, mg/dL		1.02 (0.99–1.04, *p* = 0.306)	
	TCA, mg/dL		1.13 (0.67–1.92, *p* = 0.646)	
	AST, U/L		1.01 (1.00–1.02, *p* = 0.160)	
	ALT, U/L		1.00 (0.99–1.01, *p* = 0.686)	
	LDH, U/L		1.00 (1.00–1.01, *p* = 0.330)	
	GGT, U/L		1.00 (0.99–1.00, *p* = 0.482)	
Thyroid Function Test	TSH, mIU/L		**1.04 (1.01–1.08, *p* = 0.010)**	**1.04 (1.01–1.08, *p* = 0.016)**
	fT3, pg./mL		0.74 (0.45–1.22, *p* = 0.236)	
	fT4, pmol/L		1.08 (0.99–1.16, *p* = 0.070)	
	TT3, nmol/L		0.88 (0.53–1.45, *p* = 0.617)	
	TT4, ug/dL		1.07 (0.96–1.19, *p* = 0.203)	
Urinary iodine concentration	UIC, ug/L		1.00 (1.00–1.00, *p* = 0.555)	1.00 (1.00–1.00, *p* = 0.526)
Vitamin B12 Intake	Vitamin B12 intake, mcg		0.97 (0.87–1.09, *p* = 0.604)	1.03 (0.93–1.15, *p* = 0.557)
Dietary folate Intake	Total dietary folate intake, mcg	Low group**(<**271.75mcg**)**		
		High group**(**≥271.75mcg**)**	**0.48 (0.33–0.71, *p* < 0.001)**	**0.53 (0.35–0.80, *p* = 0.003)**

n, number of patients; BMI, Body mass index; WBC, White blood cell count; HGB, Hemoglobin; RBC, Red blood cell count; NE, Segmented neutrophils num; LYM, Lymphocyte number; MO, Monocyte numbe; EO, Eosinophils number; PLT, Platelet count; RDW, Red cell distribution width; ALB, Albumin; GLO, Globulin; TP, Total protein; CHOL, Cholesterol; SCR, Creatinine; SGL, Glucose, serum; STB, Total bilirubin; TG, Triglycerides; UA, Uric acid; NA, Sodium; KS, Potassium; CL, Chloride; BUN, Blood urea nitrogen; TCA, Total calcium; AST, Aspartate aminotransferase; ALT, Alanine aminotransferase; LDH, Lactate dehydrogenase; GGT, Gamma glutamyl transferase; TSH, Thyroid stimulating hormone; fT3, Triiodothyronine (T3), free; fT4, Thyroxine, free; TT3, Triiodothyronine, total; TT4, Thyroxine, total; UIC, Urinary iodine concentration. The bold values indicate statistical significance (*p*-value < 0.05).

*Demographics*: Age showed a significant correlation with the development of AIT. Univariate logistic regression analysis revealed that for each 1-year increase in age, the risk of developing AIT increased by 1% (OR = 1.01, 95%CI: 1.00–1.02, *p* = 0.028). Multivariate analysis indicated an OR of 1.01 (95%CI: 1.00–1.02, *p* = 0.04), suggesting that the association between age and the risk of AIT remained significant after adjusting for other variables. Among patients with AIT, women are more common. In the univariate analysis, the risk of developing AIT in females was approximately 1.87 times higher than in males (OR = 1.87, 95%CI: 1.25–2.80, *p* = 0.002). Multivariate analysis confirmed that, after controlling for other confounding factors, gender remained strongly linked to the likelihood of developing AIT, with females having approximately 1.63 times higher risk than males (OR = 1.63, 95%CI: 1.07–2.47, *p* = 0.021).

*Complete blood count*: No statistically significant differences were observed in complete blood count parameters between the AIT and non-AIT groups in the univariate analysis.

*Blood biochemical indicators*: No statistical differences were found in the blood biochemical parameters between the AIT group and the non-AIT group in the univariate analysis.

*Thyroid function tests*: The average TSH level in the AIT group was significantly higher than that in the non-AIT group. The univariate analysis demonstrated that for every 1 mIU/L increase in TSH, the risk of developing AIT increased by 4% (OR = 1.04, 95%CI: 1.01–1.08, *p* = 0.01). The multivariate analysis revealed that for each 1 mIU/L increase, the risk of developing AIT rose by 4% (OR = 1.04, 95%CI: 1.01–1.08, *p* = 0.016), indicating a strong positive relationship between TSH levels and the likelihood of AIT.

*Urinary iodine concentration*: No statistical differences were found in UIC between the AIT group and the non-AIT group in the univariate analysis.

*Vitamin B12 Intake*: No statistical differences were found in vitamin B12 intake between the AIT group and the non-AIT group in the univariate analysis.

*Dietary folate intake*: Participants in the high dietary folate intake group showed a 52% lower risk of AIT compared to the low-intake group (adjusted OR = 0.48, 95%CI: 0.33–0.71, *p* < 0.001) in the univariate analysis. This association remained significant after multivariable adjustment (adjusted OR = 0.53, 95%CI: 0.35–0.80, *p* = 0.003).

In conclusion, advanced age, female sex, elevated TSH levels, and lower dietary folate intake are independent risk factors for AIT.

### Predicting the risk of AIT

The regression model for predicting AIT risk based on dietary folate intake, TSH, sex, age, UIC, vitamin B12 intake is presented below:

Logit(P) = − 0.635 × dietary folate intake (mcg/day) + 0.042 × TSH (mIU/L) + 0.488 × gender (female = 1, male = 0) + 0.011 × age (years)–0.0002 × UIC (μg/L) + 0.033 × vitamin B12 intake (mcg/day)–3.141 (constant).

By integrating the above variables, we constructed a personalized nomogram for assessing AIT risk (Figure 2B). In this nomogram, the value of each variable corresponds to a specific score on the horizontal axis, which reflects the contribution of that variable to the overall event risk. By summing the scores of each variable, the total score can be obtained. The last row of the nomogram displays the estimated likelihood corresponding to the total score, visually reflecting the individual’s risk level of developing AIT.

## Discussion

This cross-sectional study explored the association between folate consumption and the risk of developing AIT for the first time, by analyzing data from the NHANES database. The results show that dietary folate intake is negatively correlated with the likelihood of developing AIT, and lower folate intake is an independent risk factor for AIT.

Our study suggests that dietary folate intake may exert a protective effect against AIT. Likewise, previous studies have reported an inverse association between serum folate levels and the risk of other autoimmune diseases, such as vitiligo (33). However, the relationship between folate and thyroid function remains controversial. Although some studies suggest that folate may have a protective effect, research on the relationship between dietary folate intake and AIT remains limited, and further exploration in this area is needed.

In this study, the proportion of females and the average age in the AIT group were significantly higher compared to the non-AIT group. Similar findings have been reported in several previous studies (34–36), which collectively reinforce the higher incidence of AIT in females and suggest that the risk of AIT may rise with age. This could be associated with immune system aging, which typically involves an increased production of autoantibodies and a state of chronic low-grade inflammation, thereby contributing to the development of AIT (37).

Numerous studies have suggested a potential link between excessive iodine intake and the onset of AIT, possibly mediated by immune system activation mechanisms (20). Vitamin B12 and folate collaboratively contribute to Hcy metabolism in the human body. A deficiency in vitamin B12 may impair folate utilization by disrupting its metabolic pathway. Research has demonstrated a significant association between serum vitamin B12 concentrations and the incidence of AITDs, with an inverse correlation observed between B12 levels and TPOAb titers (30). In order to reduce confounding bias when examining the association between dietary folate intake and the risk of AIT, it is essential to control for iodine and vitamin B12 as potential confounding variables in this study. Univariate analysis demonstrated no significant differences in urinary iodine levels or vitamin B12 intake between the AIT and non-AIT groups. These results indicate that, after adjusting for iodine status and vitamin B12 intake, the association between dietary folate intake and the risk of AIT remains significant.

Besides, in contrast to some previous studies (35, 36), no significant differences were observed in the blood biochemical indicators between the AIT and non-AIT groups in this study. This may be due to the possibility that these biochemical markers do not serve as mediators or regulatory factors through which dietary folate intake influences AIT risk, or that the impact of folate on these parameters is too subtle to achieve statistical significance.

Folate is an essential micronutrient that, as a substrate for nucleotide synthesis and a methyl donor in the one-carbon metabolism pathway (38), plays a role in DNA synthesis, repair, methylation, and antioxidant defense. These processes are crucial for normal cell proliferation. Additionally, folate plays an important role in the regulation of immune function (39). Studies have shown that folate is crucial for the survival of regulatory T cells (Tregs) in the small intestine and can influence the composition of the gut microbiota (40–43), suggesting a potential regulatory role for folate in autoimmunity and chronic inflammation (44). A growing body of evidence highlights the critical role of the gut microbiota in the pathogenesis of AIT (9, 10), suggesting that folate deficiency may contribute to the onset and progression of AIT by altering the gut microbiota composition. Folate is also crucial for the production and metabolism of antibodies, and adequate folate levels are necessary for generating appropriate antigen-specific antibody responses (45). Folate deficiency can also lead to elevated Hcy levels. Hcy has pro-inflammatory and immune-regulatory properties, and studies suggest that Hcy may trigger autoimmunity by binding to and structurally modifying specific proteins (46). One study demonstrated that folate supplementation reduced oxidative stress and HHcy in hypothyroid rats. If verified in humans, this could offer scientific support for using folate as an adjunctive therapy for hypothyroidism (47). A recent cross-sectional study indicated that patients with HHcy had notably higher TSH levels and an increased positive rate of TPOAb (48). Nedrebo et al. found no association between plasma Hcy levels and serum folate in hypothyroid patients (49), while Barbe et al. reported a generally inverse correlation between folate and Hcy levels (50). Similar to Barbe et al., the study by Bilgin Özmen et al. also suggested that elevated Hcy levels in hypothyroid patients may be attributed to low folate levels. This indicates that changes in Hcy could result from alterations in folate status or the activity of folate-metabolizing enzymes (29). It can be inferred that folate may play a role in preventing and treating thyroid dysfunction and AIT by lowering Hcy levels.

According to the Dietary Reference Intakes (DRIs), the recommended daily folate intake for males and females aged 19 and older is 400 μg/day, for pregnant women is 600 μg/day, and for lactating women is 500 μg/day. Analyzing dietary folate intake from the NHANES 2009–2010 dataset revealed that, despite the mandatory folic acid fortification policy in the United States, individuals with AIT consumed significantly less dietary folate than those without AIT. Moreover, the mean intake in both groups remained below the recommended 400 μg of dietary folate equivalents (DFE) per day. Although this study estimated folate intake using a 24-h dietary recall method, which only accounted for folate from natural foods and fortified foods and did not include additional intake from dietary supplements, the results still suggest that individuals with AIT may have overall insufficient folate intake or differences in folate absorption and metabolism. Insufficient folate intake could potentially exacerbate the autoimmune progression of thyroid disease through several mechanisms, such as elevating homocysteine levels, which may promote inflammation and autoimmunity, impairing the function of Tregs, altering gut microbiota composition, and increasing oxidative stress. In conclusion, these results indicate that dietary folate intake could potentially influence the onset and progression of AIT, highlighting the need for further validation through longitudinal studies. However, with the gradual increase in the use of folic acid supplements, the daily folate intake of certain populations has exceeded the recommended intake levels (51). Excessive folate intake has gradually become a new health concern due to its potential negative effects on the body, such as masking clinical symptoms of vitamin B12 deficiency, increasing the risk of cancer, and contributing to birth defects in offspring (52, 53). Therefore, maintaining appropriate folate levels is crucial for normal physiological function.

This study is the first to explore the association between dietary folate intake and AIT, and the results suggest that lower dietary folate intake may be an independent risk factor for AIT. Participants were categorized into low and high folate intake groups. Following adjustment for confounders including age, sex, urinary iodine concentration, and vitamin B12 intake, the high folate intake group exhibited a significantly reduced risk of AIT compared to the low intake group. These findings imply that sufficient dietary folate intake might offer a protective effect against AIT, indicating that optimizing folate intake might represent a promising avenue for prevention. This provides preliminary guidance for further studies exploring dietary strategies aimed at reducing AIT risk. Folate may be involved in the development of AIT through mechanisms such as modulating immune system function and exerting antioxidant effects. However, the cross-sectional design of this study limits the ability to establish causality. Although we observed a negative correlation between the two, it cannot be determined whether folate deficiency directly causes AIT. At the same time, the study highlights the importance of adequate folate intake and warns of the potential health risks associated with excessive supplementation. Future longitudinal studies are needed to further validate the causal relationship between folate and AIT, as well as its potential mechanisms. Furthermore, randomized controlled trials targeting high-risk populations for AIT, such as TPOAb-positive women, are recommended to clarify the actual clinical efficacy of folate supplementation in reducing AIT incidence. Conducting dose–response studies will help identify the optimal folate supplementation protocol, thereby offering stronger evidence to support the formulation of dietary recommendations for AIT prevention.

## Limitations

First, as a cross-sectional study, it cannot determine the causal relationship between dietary folate intake and the risk of AIT. The potential for reverse causality between AIT and dietary folate intake should be acknowledged, since AIT could influence dietary habits and nutrient consumption, potentially leading to lower folate intake. Second, dietary folate data were obtained through the 24-h recall method, which may be subject to recall bias and have limitations in assessing intake of folate over time. Third, folate deficiency may result from factors such as alcohol consumption, smoking, the use of certain medications (e.g., anti-inflammatory and antiepileptic drugs), chronic diseases (e.g., liver disease), malabsorption disorders (e.g., gastrointestinal conditions or post-surgical changes), and genetic mutations (e.g., MTHFR gene variants). Additionally, aging can impair folate absorption. These factors contributing to folate deficiency may, to some extent, impact the reliability of our results. Finally, the lack of thyroid ultrasound data could potentially affect the diagnostic accuracy of AIT. Further validation is needed in the future through longitudinal studies, expanding the sample size and controlling for confounders.

## Conclusion

After analyzing the NHANES database, we identified an inverse relationship between dietary folate intake and the likelihood of developing AIT, with individuals in the low-intake group demonstrating a significantly increased risk of developing AIT compared to those in the high-intake group. The findings indicate that insufficient folate intake could be an independent risk factor for AIT. Further longitudinal research is required to better understand the causal relationship between folate and AIT, as well as the underlying mechanisms. Furthermore, rigorously designed randomized controlled trials targeting populations at high risk for AIT would be valuable to provide more definitive evidence in support of dietary prevention approaches.

## Data Availability

The original contributions presented in the study are included in the article/Supplementary material, further inquiries can be directed to the corresponding authors.

## Footnotes

^1^ https://wwwn.cdc.gov/nchs/nhanes/Default.aspx

^2^ http://www.R-project.org

^3^ https://ggplot2.tidyverse.org/
